# The Effect of Immediate Kangaroo Mother Care During Third Stage of Labor on Postpartum Blood Loss and Uterine Involution: A Quasi-Experimental Comparative Study

**DOI:** 10.3390/healthcare12242548

**Published:** 2024-12-17

**Authors:** Wedad M. Almutairi, Dareen K. Raidi

**Affiliations:** 1 Maternity and Child Department, Faculty of Nursing, King Abdulaziz University, Jeddah 22254, Saudi Arabia; 2 Nursing College, Bisha University, Bisha 67714, Saudi Arabia; dooly_1412@hotmail.com

**Keywords:** KMC, third stage of labor, postpartum blood loss, uterine

## Abstract

Background: Obstetric hemorrhage is the leading cause of maternal death worldwide. Obstetric hemorrhage accounts for 27.1% of all maternal death worldwide. Of all obstetric hemorrhages, postpartum hemorrhage (PPH) accounts for 72%. The physiological management of the third stage of labor is a growing area as a preventive measure to control postpartum blood loss. Immediate kangaroo mother care (KMC) is suggested as one of the physiological management methods of the third stage of labor to reduce postpartum blood loss. The duration of the third stage of labor, uterine involution, and amount of postpartum blood loss are the physiological parameters of effective management of the third stage of labor. Examining the absolute effects of immediate KMC on maternal physiological parameters is needed in different populations with different settings. Thus, this study aimed to examine the effects of immediate KMC on uterine involution and postpartum blood loss. Methods: A quasi-experimental comparative design was conducted in the labor and delivery room at Maternity and Children Hospital, Makkah, Saudi Arabia. A sample of 80 women was divided into two equal groups: a treatment group that underwent immediate KMC and a control group that received routine care. Instrument: A questionnaire developed by the researchers was used to collect the data. Results: The effects of immediate KMC were significant concerning uterine involution and regarding the uterine position immediately after placenta separation (70% at umbilicus, χ^2^ = 8.5, *p* < 0.01), postpartum blood loss (χ^2^ = 76.098, *p* < 0.00), the heaviness of lochia (χ^2^ = 44.679, *p* = 0.00), and the number of pads used in the first 24 h (*p* < 0.001).

## 1. Introduction

Obstetric hemorrhage accounts for 27.1% of all maternal deaths, making it the leading cause of maternal mortality globally (WHO, 2020). Of all obstetric hemorrhages, postpartum hemorrhage (PPH) accounts for 72% [1]. PPH is a preventive condition when all labor stages are managed appropriately. One method for managing PPH is the active management of the third stage of labor (AMTSL), which is the most common intervention in the hospital setting. Another method is expectant management, which means a hands-off approach during the third stage of labor (TSL), which is also called physiological management when both kangaroo mother care (KMC) and breastfeeding are involved. Both management methods are presented in the literature as appropriate for PPH prevention.

AMTSL is a preventive measure for controlling postpartum blood loss for the prevention of PPH and consists of the administration of exogenous oxytocin (Pitocin), control cord traction, and early cord clamping; however, updated evidence confirms that only Pitocin administration has a significant effect on postpartum blood loss [1]. The physiological management of TSL is a growing topic as a preventive measure to control postpartum blood loss [2]. Based on Saxton’s theory, the physiological management of TSL consists of immediate KMC and breastfeeding, which are preventive measures of PPH [3]. Kangaroo mother care (KMC), especially via skin-to-skin contact (SSC) and breastfeeding (BF), significantly decreases postpartum blood loss by augmenting physiological mechanisms that facilitate uterine contraction. During SSC and BF, the maternal body secretes endogenous oxytocin, a hormone essential for stimulating uterine contractions. These contractions assist the uterus in constricting the blood arteries at the placental location, thus minimizing hemorrhage. Furthermore, the release of oxytocin promotes uterine involution, allowing the uterus to more effectively remove blood clots and the placenta, therefore reducing the duration of the third stage of labor and lowering the risk of postpartum hemorrhage [4,5].

Furthermore, skin-to-skin contact and BF play a role in the control of stress hormones in the mother. SSC improves the efficacy of oxytocin in facilitating uterine contractions and milk ejection by reducing cortisol and ACTH levels. Breastfeeding enhances oxytocin secretion via nipple stimulation, which immediately fortifies uterine tone and decreases blood loss. Clinical studies have repeatedly shown that women who participate in skin-to-skin contact (SSC) and breastfeeding (BF) within the first hour post-delivery enjoy shorter durations of the third stage of labor, dramatically reduced estimated blood loss, and a lower incidence of uterine atony, the primary cause of postpartum hemorrhage. KMC provides a natural, economical, and non-pharmacological approach to enhance maternal health and reduce the risks linked to postpartum hemorrhage [4,5].

The main objective of third stage management is to prevent PPH by shortening the duration of the TSL to less than 20 min and enhancing uterine contractility. A shorter duration of TSL, uterine involution, and the amount of postpartum blood loss are the physiological parameters of effective management of TSL [4]. Examining the absolute effects of immediate KMC on maternal physiological parameters is needed in different populations and different settings [5]. However, inadequate evidence remains regarding the effects of KMC during TSL on the mothers’ post-birth physiological parameters.

### 1.1. Purpose of the Study

The purpose of our study was to examine the effects of immediate KMC on the duration of TSL, uterine involution, and postpartum blood loss.

### 1.2. Research Hypothesis

In our study, we hypothesized that immediate KMC leads to a decrease in the duration of TSL, improves uterine involution, and minimizes postpartum blood loss within the first 24 h following birth.

Our study examined the effects of immediate KMC on the physiological parameters of postpartum blood loss, the duration of TSL, and uterine involution. The study utilized four main concepts: immediate KMC, duration of TSL, uterine involution, and postpartum blood loss.

## 2. Materials and Methods

The study followed a quasi-experimental design to fulfill the aim of our study. This study was conducted in a delivery room in the obstetrics and gynecological department in a tertiary hospital under the Ministry of Health in Makkah, Saudi Arabia. The hospital practices the principles of a baby-friendly hospital. At the time of the study, 54 nurses worked in the labor and delivery department. Our sample represents the population of women who had normal vaginal deliveries and were able to conduct immediate KMC. We used a purposive sampling method to obtain access to women who met the study inclusion criteria. Women who met the following inclusion criteria were asked to participate in the study. The inclusion criteria were women who delivered a term infant/newborn (38–42 weeks’ gestation), women who had a normal vaginal delivery, women who delivered a single viable fetus in cephalic presentation, and women willing to join the study. We excluded women who experienced premature labor (<38 weeks) or low birth weight (<2500 g); women who had a newborn with signs of respiratory distress at birth; women who had placental abnormalities or any condition leading to an over-distended uterus, such as multiple gestation or polyhydramnios, high blood pressure disorders of pregnancy; women who had a history of prolonged labor or premature rupture of membrane; and women who used forceps or vacuum-assisted delivery for their current birth. Obstetric complications can independently affect maternal outcomes, and women with obstetric complications may require additional medical interventions. This could interfere with implementing immediate KMC, which requires an alert mother to prevent any maternal or neonatal risk.

Sample size calculations generally depend on the required effect size, significance level, statistical power, and the standard deviation of the outcome variable. In a quasi-experimental study comparing two groups (e.g., intervention versus control), the formula for calculating the sample size is: N = (Zα + Zβ)2 × (2 × σ2).

Δ2

Significance level (α = 0.05): The significance level represents the probability of committing a Type I error—incorrectly rejecting a true null hypothesis. By setting α = 0.05, the study accepted a 5% chance of a false positive result. This threshold balanced rigor and practicality, as a more rigorous level (e.g., α = 0.01) would require a larger sample size, making the study less feasible. The statistical power is the probability of detecting a true effect if it exists. With β = 0.2, the study had an 80% chance of identifying a significant difference when one exists. A power of 0.80 is commonly accepted in research as it provides a good balance between detecting true effects and avoiding unnecessary resource use. Increasing power (e.g., to 90%) would require a larger sample size which might not be feasible. The total sample size after the calculation was 80 (40 for each group). The sample was divided into two groups: a control group that did not receive intervention and an intervention group that received the intervention.

### 2.1. Variables of the Study

The study included independent and dependent variables. Based on our study model, immediate KMC was the independent variable that represented the study intervention. Immediate KMC was defined as SSC between mothers and newborns during the TSL. Therefore, we operationalized the definition of immediate KMC as SSC between mothers and newborns starting from the newborn’s birth for 10 min using a stopwatch to count the duration of the immediate SSC.

The study had three main dependent variables: the duration of the TSL, uterine involution, and postpartum blood loss. The operational definition of the TSL is the number of minutes taken from newborn birth until complete birth of the placenta using a stopwatch. The operational definition of uterine involution is the characteristics of firmness, position, and level of the uterus immediately after placenta delivery, 6 h postpartum, and 24 h postpartum. The operational definition of postpartum blood loss is the documented estimated amount of blood loss, heaviness of lochia, number of pads consumed within the first 24 h, and the hemoglobin level. Data were collected from two groups of 40 women each (intervention and control groups) regarding the duration of the TSL, uterine involution, estimated amount of blood loss in milliliters, heaviness of lochia, and the number of pads consumed in the first 24 h.

### 2.2. Study Instruments

The study instrument was a measurement tool developed by the researcher to capture demographic and reproductive data. Demographic variables were age, educational level, occupation, duration of marriage, and nationality. Other variables included weight, height, BMI, and medical history.

Reproductive data included parity (number of living children); gravidity (number of pregnancies); number of abortions; number of antenatal visits; and involution assessment form, which included firmness, position, and the level of the uterus. Additionally, the type of placental delivery, completeness of placenta, duration of the TSL, and blood loss were included in the reproductive data. The assessment form included the number of pads consumed in the first 24 h, heaviness of lochia, and hemoglobin level 1-day postpartum.

### 2.3. Intervention

The researcher explained the SSC to mothers using a brochure before initiating the process. The respondents understood SSC as beginning when the baby was born, at which time the newborn would be placed directly on the mother’s chest, naked and without any blanket before the umbilical cord is cut. The head was covered with a sheet so that the body temperature was maintained until the placenta was delivered, after which the duration was measured using a stopwatch. Both groups (intervention and routine care) received international units (IU) of oxytocin administered intramuscularly per hospital guidelines.

### 2.4. Procedure

Official approval was obtained from the ethical committee in the Faculty of Nursing, King Abdulaziz University and the Maternity and Children Hospital in Makkah Al-Mukaromah. The researchers received a Bioethics Online Training Course Certificate. Approval from the director of the nursing department at the hospital was obtained. The researchers provided the director with information about the purpose of the study, and a mutually agreed upon date and time for initiating data collection for the study was selected to obtain ethical permission to conduct the quasi-experimental study. The nursing director emailed the memo to the head nurse at the selected unit to facilitate the researcher’s work. The researcher explained the purpose of the study to the study participants. Informed consent was obtained from all participants. The study was divided into three phases as follows.

#### 2.4.1. Phase I: Assessment and Planning Phase

The researcher prepared the current tool after a comprehensive review of the related literature. Participating women were recruited according to the eligibility criteria. Those who agreed to participate were divided by days into two groups of 40 women each: the first two days, the women applied KMC (intervention group); and the other two days, the women received routine hospital care (control group). The researcher explained the purpose and procedures of the study to each participant, and informed consent was obtained before data collection. The researcher asked each woman individually about her sociodemographic data. Data were collected by the researcher who was available 4 days per week in the morning shift from 8 am to 4 pm.

#### 2.4.2. Phase II: Implementation Phase

Based on the assessment phase, the researcher visited the delivery room at MCH Maternity and Children hospital in Makah and met the participants. The researcher introduced herself and explained the purpose of the study. The participants of the study were selected and divided by days into two equal groups (intervention and control groups). The tool was then completed by the researcher for all participants of both the intervention and control groups.

#### 2.4.3. Phase III: Evaluation Phase

In this phase, the participants in both groups were evaluated using the same tool immediately during the TSL, and within the first 24 h after birth. The evaluation entailed documenting changes following the execution of SSC or routine care to the experimental and control groups, respectively. The phase began when the baby was born and placed directly on the mother’s chest, naked and without any blanket, before the umbilical cord was cut. The head was covered with a sheet so that the body temperature was maintained until the placenta delivery. The duration was measured by using a stopwatch, and uterine involution was assessed and measured by the attended nurse and the researcher.

### 2.5. Statistical Analysis

Data were sorted and filtered before being entered into the IBM-SPSS for analysis. Descriptive statistics were calculated, including categorical variables described by numbers and percentages, and continuous variables described by the mean, median, minimum, maximum, and standard deviations (SD). Inferential statistics included chi-square and paired *t*-tests to infer differences between the intervention and control groups.

### 2.6. Data Management

All extracted data (hard copies) from the study were saved in a locked cabinet in the advisor’s office. All electronic data were saved on a password-protected computer so that only the researchers could access the data.

### 2.7. Ethical Considerations

Ethical approval was obtained from FON and the Ministry of Health. Informed consent was obtained from the respondents and the healthcare institutions before data collection. Informed consent ensured that participating in this study would not affect the medical care provided to the patient in any way. Confidentiality was maintained by de-identifying all the personal data, and study ID numbers were assigned to each participant enrolled in the study. All clinical data were labeled using the code number to enhance privacy and confidentiality. Participation in this study was voluntary, so that the respondents had the discretion to refuse to participate or withdraw from the study at any time without jeopardy to privileges and access to healthcare.

A fair and understandable explanation of the purposes of the research and the expected duration of participation were provided to each woman. A description of the procedures to be followed and identification of any experimental procedures were also provided. All consent forms had a signature line for the participant, and the researchers anonymized the respondents’ data to enhance confidentiality and privacy of their health status. The anonymity prevented third parties from accessing data for other use, other than the prescribed procedures of the study.

### 2.8. Validity and Reliability

In this study, face and content validity were tested. The tool was tested for its content and face validity by experts in the nursing field, and modifications were made as necessary. To ensure the reliability of this study, inter-rater reliability was established by described standards and methods, extensive training sessions, and calibration exercises for all data collectors to match their understanding of it. A preliminary investigation facilitated the identification and fixing of inconsistencies prior to comprehensive data collection, and was applied using objective tools such checklists and visual aids. This facilitated consistent decision-making, e.g., we used visual assessment measure pictures for the amount of blood loss, which have been explained and defined for all data collectors. The percentage of agreement was applied on 10 samples of data collection to ensure the evaluation consistency of the rater prior to actual data collection, and then periodic verification was applied.

In this study, the researcher and the medical professionals (nurses, doctors) who were present in the delivery room and assigned to the patient during the study had agreed on the information gathered.

## 3. Results

The study sample was 80 women who had a spontaneous vaginal delivery at the Maternity and Children Hospital in Makkah Al-Mukarramah. The largest group of participants were > 30 years old (n = 25, 31.2%), followed by 27–30 years old (n = 23, 28.7%). Over half of the sample (n = 49, 61.3%) had university and higher education. The majority of the participants were homemakers (n = 69, 86.2%) and Saudi (n = 71, 88.8%). Almost half of the participants had been married 1–3 years (n = 36, 45%). Approximately half of the sample participants (46.2%) were overweight, which was measured as a BMI of 25 ≤ 30. The majority of the participants did not have diabetes mellitus (DM; n = 79, 98.8%), and none of the study participants had hypertension, gestational DM, gestational hypertension, anemia, heart disease, urinary tract infection, or recurrent vaginal infection. Just over half of the sample had been pregnant 1–2 times (n = 45, 56.2%), with the next largest group reporting more than four pregnancies (n = 18, 22.5%). In terms of deliveries, 46.2% (n = 37) of the sample were primipara, and 42.5% (n = 34) had one to two deliveries. Most of the participants did not have abortions (n = 63, 78.8%). Half of the sample did not have previous children (n = 40, 50%), and 23.8% (n = 19) had three to four children.

### 3.1. Description of the Study Variables

Duration of the Third Stage: The majority of women who had immediate KMC (n = 34, 85%) had a TSL lasting 5 min to less than 10 min compared to only six women (15%) from the control group. Most of the women in the control group (n = 34, 85%) had a TSL lasting 10 to 20 min. Postpartum Blood Loss: Regarding the heaviness of lochia, 67.5% (n = 27) of the women in the intervention group had light lochia, whereas 57.5% (n = 23) of the women in the control group had moderate lochia 24 h postpartum. Regarding the number of pads consumed in the first 24 h, the result showed a significant difference in the mean score between both groups. Uterine Involution: Regarding the position of the uterus, most of the women in the intervention (n = 36, 90%) and control (n = 33, 82%) groups had their uterus at the middle immediately after placenta separation. Likewise, the majority of women in the SSC (n = 32, 80%) and control groups (n = 36, 90%) had their uterus at the middle after 6 h. Concerning the level of the uterus, the majority of women in the SSC group (n = 26, 65%) had their uterus one and two fingers below the umbilicus immediately after placenta separation and at 6 h, whereas the majority of women in the control group (n = 28, 70%) did not. Concerning the level of the uterus 24 h after placenta separation, the majority of women in the control group had their uterus one finger below the umbilicus (n = 28, 70%), whereas most women (n = 26, 65%) in the SSC group did not. However, the majority of women in the SSC group had their uterus two fingers below the umbilicus after 24 h (n = 26, 65%), whereas most women (n = 28, 70%) in the control group did not. Number of Minutes of Immediate Kangaroo Mother Care: all study participants received the study intervention within a maximum of 10 min after delivery.

### 3.2. Demographic Characteristics Among Study Groups

Table 1 shows the distribution of the study participants according to their age groups, educational level, and occupation. The table shows that the highest percentage (n = 28, 35.0%) in the intervention group were aged between 27 and 30 and 30 years or older. However, in the control group, women aged 23–26 years accounted for the highest percentage (n = 15, 37.5%). Regarding educational level, the highest percentage of participants in the intervention group (n = 23, 57.5%) and control group (n = 20, 50.0%) had a university education and higher. Concerning participants’ occupation, the highest percentages of participants in the SSC and control groups were homemakers (n = 37, 92.5% and n = 32, 80.0%, respectively).

Table 2 below shows that most women in the SSC group (n = 16, 40.0%) have been married more than 6 years, whereas, in the control group, the majority (n = 22, 55.0%) have been married 1–3 years. Regarding sample nationality, the majority were Saudi in both the SSC and control groups (n = 37, 92.5% and n = 34, 85.0%, respectively). Concerning BMI level, most women in both the SSC and control groups were overweight (n = 20, 50.0% and n = 17, 42.5%, respectively).

Table 3 shows that half of the mothers in the SSC (n = 20, 50.0%) and control groups (n = 20, 50.0%) had one or two pregnancies. Furthermore, half of the SSC group (n = 20, 50.0%) had one to two deliveries, whereas just over half of the mothers in the control group (n = 22, 55.0%) had yet to give birth. Regarding abortions, the majority of the SSC group (n = 34, 85.0) indicated that they had never had an abortion. However, 40% (n = 16) of the mothers in the control group indicated that they had three to four abortions. Moreover, concerning the number of children, the majority of participants in both the SSC and control groups indicated that they did not have children (n = 15, 37.5% and n = 25, 62.5%, respectively).

### 3.3. Results of the Analysis of the Research Questions

#### 3.3.1. RQ1: What Is the Effect of Immediate KMC on Uterine Involution?

Uterine involution was measured by uterine firmness, position, and levels. Regarding uterine firmness, all of the women in the SSC and control groups had uterus firmness immediately after placenta separation. Table 4 shows that regarding uterine position, no significant association was found between SSC and the position (middle, right, and left; *p* > 0.05) immediately and after 6 h. Regarding uterine levels, a significant association was found between SSC and the level of the uterus (at the umbilicus, one and two fingers below umbilicus) immediately after placenta separation and after 6 h (*p* < 0.05) (Table 5).

Regarding the uterus level, Table 6, Table 7 and Table 8 report a significant association between SSC and the level of the uterus (at the umbilicus and one and two fingers below the umbilicus) immediately after placenta separation and after 6 h (*p* < 0.05). The post hoc showed that SSC had a significant positive effect on uterine position (at the umbilicus and one finger below the umbilicus) immediately after placenta separation. In addition, the post hoc showed that SSC had a significant positive effect on the uterine position (at and below the umbilicus) after 6 h. Moreover, a significant association was found between SSC and the level of the uterus (one and two fingers below the umbilicus) after 24 h (*p* < 0.05). The post hoc also showed that SSC had a significant positive effect on the uterine position (one and two fingers below the umbilicus) after 24 h.

#### 3.3.2. RQ2: What Is the Effect of SSC on Postpartum Blood Loss?

Postpartum blood loss was measured by the heaviness of lochia, the number of pads consumed in the first 24 h, and hemoglobin level at 1-day postpartum. Regarding the heaviness of lochia, a significant association was found between SSC and the heaviness of lochia (χ^2^ = 44.679, *p* < 0.05) (Table 9). A post hoc analysis showed that SSC had a significant effect on the reduction of lochia post-delivery.

Table 10 demonstrates the effect of SSC on the number of pads consumed in the first 24 h and the hemoglobin level on the first day postpartum. The mean number of pads consumed in the first 24 h was significantly different between the groups (*p* < 0.001), with women in the SSC group using significantly fewer pads than those in the control group. Concerning hemoglobin level on the first day postpartum, no significant difference was found between hemoglobin levels on the first day postpartum between both groups (*p* > 0.05).

### 3.4. Effect of Skin-to-Skin Contact on Duration of Third Stage of Labor

Table 11 shows a significant association between SSC and the duration of TSL (*p* < 0.05). The post hoc analysis showed that SSC had a significant effect on the reduction of the duration of TSL.

## 4. Discussion

### 4.1. Effect of Skin-to-Skin Contact on Uterine Involution

Our study demonstrated that immediate skin-to-skin contact (SSC) significantly impacts uterine involution, mainly in the positioning and level of the uterus within the first 24 h postpartum. The findings align with recent research emphasizing the role of SSC in decreasing the fundal height and accelerating uterine involution through oxytocin stimulation [6]. Similarly, early SSC enhances uterine contractility, a critical factor in expediting involution [7]. However, while this study observed that the uterus was predominantly established at the middle position postpartum, other studies noted significant reductions in fundal height, suggesting that fundal measurements may offer additional clarity in future studies [6].

In contrast to the findings regarding the effects of SSC on uterine position [8], this study found notable differences, especially in uterine levels immediately after and 6 h post-placenta separation. Such variability underscores the potential influence of contextual factors, such as sample demographics or the timing of SSC initiation [9,10,11].

### 4.2. Effect of Skin-to-Skin Contact on Postpartum Blood Loss

The results indicated that SSC significantly reduces postpartum blood loss, evidenced by a decrease in lochia severity and reduced consumption of sanitary pads [6,7]. This reduction is likely mediated by enhanced oxytocin release during SSC, which facilitates stronger uterine contractions. Furthermore, managing postpartum anemia, a risk moderated through SSC by reducing hemorrhage severity and stabilizing hemoglobin levels, has been emphasized [12]. In contrast, other research reported no significant reduction in lochia volume, suggesting that SSC’s impact may vary due to differences in methodological approaches or participant compliance with SSC protocols [6,13,14].

### 4.3. Effect of Skin-to-Skin Contact on the Duration of the Third Stage of Labor

The study identified a significant reduction in the duration of the third stage of labor (TSL) among participants who engaged in SSC, consistent with findings highlighting SSC’s ability to expedite placental expulsion [8]. The physical stimulation provided by neonatal movements on the maternal chest likely contributes to this effect, as outlined in prior research [9]. While this study aligns with systematic reviews that SSC accelerates TSL [7], variability in effect is noted across studies, with some observing no significant acceleration, suggesting that SSC’s impact on placental separation may depend on maternal parity or other individual factors [6,15,16].

## 5. Limitations

This study has many limitations that may affect the generalizability of the results. The limited sample size in the study weakens the statistical power, constrains the capacity to identify significant differences or correlations among variables, and diminishes the representativeness of the study population. Secondly, data collection occurred in a singular setting, potentially limiting the generalizability of the findings to alternative contexts or groups. Divergences in healthcare procedures, resources, and cultural factors across different contexts may restrict the generalizability of the findings to other hospitals, regions, or demographic cohorts. Subsequent research should focus on incorporating bigger, more heterogeneous samples across other contexts to improve the robustness and external validity of the findings.

### 5.1. Clinical and Research Implications and Recommendation of the Study

#### 5.1.1. Clinical Implications

The findings of this study can be used to inform healthcare providers about the positive impacts of KMC on a mother’s maternal outcomes, such as reducing the duration of the TSL and the risk of postpartum blood loss, and encouraging uterine involution. Therefore, they should consider the application of this cost-effective, non-pharmacological intervention into their routine for immediate post-delivery care. However, as this study is a quasi-experiment and lacks randomization, RCTs are needed to confirm the positive effect of applying KMC.

#### 5.1.2. Research Implications

KMC provides encouraging findings for recommending further study to establish its influences and connections with reduced incidence of PPH, postpartum depression, and improved birth satisfaction in both normal and operative delivery. Future RCTs with control over confounders in each group, such as sociodemographic variables, are recommended. Moreover, conducting a longitudinal prospective study to determine the impact of applying KMC on both maternal and infant outcomes is recommended. Furthermore, conducting an educational need assessment to assess nursing and mothers’ knowledge, attitudes, and practice of KMC is needed to inform the development of continuous education and training programs and enhance the implementation of this critical intervention.

## 6. Conclusions

Skin-to-skin contact has a significant role in uterine involution represented by the uterus position immediately after birth and after 24 h. Skin-to-skin contact was confirmed to be of use in physiological management to minimize the amount of postpartum blood loss and significantly decrease the duration of the third stage of labor. Skin-to-skin contact is considered a cost-effective practice to improve the quality of postpartum care.

## Figures and Tables

**Table 1:** Distribution of socio-demographic characteristics for both skin-to-skin and control groups. (n = 80).

Variables	Skin-to-Skin Group n (%)	Control Group n (%)	Total n (%)
**Age Group**			
18–22 years	5 (12.5)	5 (12.5)	10 (12.5)
23–26 years	7 (17.5)	15 (37.5)	22 (27.5)
27–30 years	14 (35.0)	9 (22.5)	23 (28.7)
>30 years	14 (35.0)	11 (27.5)	25 (31.2)
**Education Level**			
Illiterate	2 (5.0)	3 (7.5)	3 (3.8)
Primary–intermediate	6 (15.0)	5 (12.5)	6 (7.5)
Secondary diploma	20 (50.0)	12 (30.0)	22 (27.5)
University and more	23 (57.5)	20 (50.0)	49 (61.3)
**Occupation**			
Student	1 (2.5)	2 (5.0)	3 (3.8)
Working	2 (5.0)	6 (15.0)	8 (10.0)
Housewife	37 (92.5)	32 (80.0)	69 (86.2)

**Table 2:** Sample distribution according to the groups’ duration of marriage, nationality, and BMI (n = 80).

Variables	Skin-to-Skin Group n (%)	Control Group, n (%)	Total n (%)
**Duration of marriage**			
1–3 years	14 (35.0)	22 (55.0)	36 (45.0)
4–6 years	10 (25.0)	9 (22.5)	19 (23.8)
>6 years	16 (40.0)	9 (22.5)	25 (31.2)
**Nationality**			
Saudi	37 (92.5)	34 (85.0)	71 (88.8)
Non-Saudi	3 (7.5)	6 (15.0)	9 (11.2)
**BMI**			
Normal (18.5 ≤ 25.0)	5 (12.5)	7 (17.5)	12 (15.0)
Overweight (25.0 ≤ 30.0)	20 (50.0)	17 (42.5)	37 (46.2)
Obese (≥30.0)	15 (37.5)	16 (40.0)	31 (38.8)

**Table 3:** Sample distribution according to reproductive data of the study groups (n = 80).

Reproductive Data	Skin-to-Skin Group n (%)	Control Group, n (%)	Total n (%)
**Gravida**			
1–2	20 (50.0)	20 (50.0)	45 (56.2)
3–4	9 (22.5)	8 (20.0)	17 (21.2)
>4	11 (27.5)	7 (17.5)	18 (22.5)
**Parity**			
None	15 (37.5)	22 (55.0)	37 (46.2)
1–2	20 (50.0)	14 (35.0)	34 (42.5)
3–4	5 (12.5)	4 (10.0)	9 (11.2)
**Abortion**			
None	34 (85.0)	10 (25.0)	63 (78.8)
1–2	5 (12.5)	1 (2.5)	15 (18.8)
3–4	1 (2.5)	16 (40.0)	2 (2.5)
**Children**			
None	15 (37.5)	25 (62.5)	40 (50.0)
1–2	6 (15.0)	6 (15.0)	12 (15.0)
3–4	13 (32.5)	6 (15.0)	19 (23.8)
≥5	6 (15.0)	3 (7.5)	9 (11.2)

**Table 4:** Effect of skin-to-skin contact on position of the uterus immediately after placenta separation (80).

Variable	Skin-to-Skin Group n (%)	Control Group, n (%)	Total n (%)	Chi-Square	*p*-Value
**Uterus at the Middle Immediately After Placenta Separation**					
Yes	33 (82.5)	36 (90.0)	69 (68.2)	0.949	0.330
No	7 (17.5)	4 (10.0)	11(13.8)		
**Uterus at the Right Immediately After Placenta Separation**					
Yes	4 (10.0)	3 (7.5)	7 (8.8)	0.157	0.692
No	36 (90.0)	37 (92.5)	73 (91.2)		
**Uterus at the Left Immediately After Placenta Separation**					
Yes	3 (7.5)	1 (2.5)	4 (5.0)	1.053	0.305
No	37 (92.5)	39 (97.5)	76 (95.0)		

Chi-square test.

**Table 5:** Effect of skin-to-skin contact on position of the uterus after 6 h.

Variable	Skin-to-Skin Group n (%)	Control Group, n (%)	Total n (%)	Chi-Square	*p*-Value
**Uterus at the Middle After 6 h**					
Yes	32 (80.0)	36 (90.0)	68 (85.0)	1.569	0.210
No	8 (20.0)	4 (10.0)	12 (15.0)		
**Uterus at the Right After 6 h**					
Yes	4 (10.0)	3 (7.5)	7 (8.8)	0.157	0.692
No	36 (90.0)	37 (92.5)	73 (91.2)		
**Uterus at the Left After 6 h**					
Yes	3 (7.5)	1 (2.5)	4 (5.0)	1.053	0.305
No	37 (92.5)	39 (97.5)	76 (95.0)		

Chi-square test, Fisher’s exact test.

**Table 6:** Effect of skin-to-skin contact on the level of the uterus immediately after placenta separation.

Variable	Skin-to-Skin Group n (%)	Control Group, n (%)	Total n (%)	Chi-Square	*p*-Value
**Uterus at Umbilicus Immediately After Placenta Separation**					
Yes	15 (37.5)	28 (70.0)	43 (53.8)	8.498	0.004
No	25 (62.5)	12(30.0)	37 (46.2)		
**Uterus One Finger Below Umbilicus Immediately After Placenta Separation**					
Yes	26 (65.0)	12 (30.0)	38 (47.5)	9.825	0.002
No	14 (35.0)	28 (70.0)	42 (52.5)		

Chi-square test, Fisher’s exact test.

**Table 7:** Effect of skin-to-skin contact on the level of the uterus after 6 h.

Variable	Skin-to-Skin Group n (%)	Control Group, n (%)	Total n (%)	Chi-Square	*p*-Value
**Uterus at Umbilicus after 6 h**					
Yes	14 (35.0)	28 (70.0)	42 (52.5)	9.825	0.002
No	26 (65.0)	12 (30.0)	38 (47.5)		
**Uterus One Finger Below Umbilicus After 6 h**					
Yes	26 (65.0)	12 (30.0)	38 (47.5)	9.825	0.002
No	14 (35.0)	28 (70.0)	42 (52.5)		
**Uterus Two Fingers Below Umbilicus After 6 h**					
Yes	26 (65.0)	12 (30.0)	38 (47.5)	9.825	0.002
No	14 (35.0)	28 (70.0)	42 (52.5)		

Chi-square test, Fisher’s exact test.

**Table 8:** Effect of skin-to-skin contact on the level of the uterus after 24 h.

Variable	Skin-to-Skin Group n (%)	Control Group, n (%)	Total n (%)	Chi-Square	*p*-Value
**Uterus One Finger Below Umbilicus After 24 h**					
Yes	14 (35.0)	28 (70.0)	42 (52.5)	9.825	0.002
No	26 (65.0)	12 (30.0)	38 (47.5)		
**Uterus Two Fingers Below Umbilicus After 24 h**					
Yes	26 (65.0)	12 (30.0)	38 (47.5)	9.825	0.002
No	14 (35.0)	28 (70.0)	42 (52.5)		

Chi-square test, Fisher’s exact test.

**Table 9:** Effect of skin-to-skin contact on postpartum blood loss and heaviness of lochia.

Blood Loss	Skin-to-Skin Group n (%)	Control Group, n (%)	Total n (%)	Chi-Square	*p*-Value
**Heaviness** **of Lochia**					
Light	27 (67.5)	0 (0.0)	27 (33.8)	44.679	0.000
Moderate	12 (30.0)	23 (57.5)	35 (43.8)		
Heavy	1 (2.5)	17 (42.5)	18 (22.5)		

Chi-square test, Fisher’s exact test.

**Table 10:** Effect of skin-to-skin contact on the number of pads consumed in the first 24 h and hemoglobin level on the first day postpartum.

Variable	N	Mean	SD	t (df)	*p* Value *
***Number of pads consumed in the first 24 h***					
Skin-to-skin group	40	2.40	0.08	−8.408 (78)	0.000
Control group	40	3.37	0.07		
***Hemoglobin level in the day 1 postpartum***					
Skin-to-skin group	40	11.74	1.26	1.564 (78)	0.122
Control group	40	11.23	1.65		

* Independent sample *t* test.

**Table 11:** Effect of skin-to-skin contact on duration of third stage of labor.

Variable	Skin-to Skin Group n (%)	Control Group, n (%)	Total n (%)	Chi-Square	*p*-Value
**Duration of Third Stage of Labor**					
5 ≤ 10 min	34 (85.0)	6 (15.0)	40 (50.0)	42.164	0.000
10 ≤ 20 min	5 (12.5)	34 (85.0)	39 (48.8)		
>30 min	1 (2.5)	0 (0.0)	1 (1.2)		
Total	40 (100.0)	40 (100.0)	80 (100.0)		

Fisher’s exact test.

## Data Availability

All related data are presented in the article.
